# Knowledge, attitudes, and practices of pharmacists towards chronic kidney disease in Rawalpindi and Islamabad, Pakistan: a cross-sectional study

**DOI:** 10.1186/s12909-026-09070-5

**Published:** 2026-03-24

**Authors:** Muhammad Amir Hamza, Hamza Siddique, Shahan Ullah, Shairyar Afzal, Mariam Masud, Mudassar Iqbal Arain, Ali Ahmed

**Affiliations:** 1 Department of Pharmacy Practice, Faculty of Pharmaceutical Sciences, Riphah Institute of Pharmaceutical Sciences, Riphah International University, Islamabad, Pakistan; 2 Department of Pharmacy Practice, Faculty of Pharmaceutical Sciences, Lahore University of Biological and Applied Sciences (UBAS), Lahore, Pakistan; 3 Department of Pharmacy, DHQ Hospital Jhelum, Jhelum, Pakistan; 4 Department of Nephrology, Pakistan Institute of Medical Science (PIMS), Islamabad, Pakistan; 5 Clinical Research Program, School of Nursing, College of Health and Human Services, University of North Carolina Wilmington (UNCW), Wilmington, NC USA; 6 Division of Infectious Diseases and Global Public Health, School of Medicine, University of California, San Diego (UCSD), La Jolla, CA USA

**Keywords:** Pharmacist role, CKD awareness, Kidney disease management, Medication counseling, Pakistan healthcare

## Abstract

**Background:**

Chronic kidney disease (CKD) is a significant public health concern, primarily affecting older adults and individuals with comorbidities such as diabetes and hypertension. The presence of multiple comorbidities often leads to polypharmacy, thereby increasing the risk of complex drug-drug and drug-disease interactions. Pharmacists play a pivotal role in minimizing these risks and ensuring safe and appropriate therapy and care for patients with CKD. This study aimed to evaluate hospital and community pharmacists’ knowledge, attitudes, and practices (KAP) regarding CKD and identify the perceived barriers to CKD care.

**Methods:**

A descriptive cross-sectional study was conducted from September 2023 to January 2024 in Rawalpindi and Islamabad, Pakistan. Hospital and community pharmacists were recruited via non-probability convenience and snowball sampling. A self-administered exploratory, context-specific questionnaire was used to collect data from 406 participants. Data were analyzed using SPSS version 23.0, employing both descriptive and inferential statistics, including Spearman correlation and logistic regression.

**Results:**

Of the 432 respondents, 26 were excluded due to incomplete data, resulting in a valid response rate of 93.9% (406). Slightly more than half of the respondents were female (51.5%), nearly half were aged 22–30 years (48.0%), and the majority had 1–5 years of experience (68.7%). Median KAP scores were: knowledge 12 (IQR10-14), attitude 34 (IQR 31–37), and practice 24 (IQR 21–26). Good knowledge, positive attitude, and good practice were observed in (57.4%), (53.0%), and (50.2%) of respondents, respectively. Regarding barriers, limited follow-up after consultation (50.0%) and lack of awareness among patients/community (45.8%) were the most frequently reported. Significant positive correlations were found between knowledge and attitude (*r* = 0.207), knowledge and practice (*r* = 0.237), and attitude and practice (*r* = 0.472) (all *p* < 0.001). Binary logistic regression revealed that training predicted higher knowledge, while practice location and seminar participation predicted positive attitude and good practice (all *p* < 0.05).

**Conclusions:**

Gaps exist in pharmacists’ CKD-related KAP, highlighting the need for targeted education and training programs. Strengthening pharmacy curricula and providing ongoing professional development are crucial to enhancing pharmacists’ clinical competencies and improving care for CKD.

**Supplementary Information:**

The online version contains supplementary material available at 10.1186/s12909-026-09070-5.

## Background

Chronic kidney disease (CKD) is a significant public health issue impacting roughly 8–16% of the global population [1–3]. The mortality rate linked with CKD is projected to increase to 14 per 100,000 individuals by 2030 [4]. Despite improvements in healthcare, developing nations continue to experience a rapid rise in CKD incidence and mortality, often at rates several times higher than in developed countries [5]. In Pakistan, the estimated prevalence of CKD ranges from 12.5% to 31.2% depending on study populations and methodologies, with an overall pooled estimate of around 21.0% [6–8]. CKD patients are often exposed to polypharmacy and complex treatment regimens due to advanced age and comorbidities, such as hypertension, diabetes, and cardiovascular complications. This increases the likelihood of drug-related problems (DRPs), including inappropriate dosing, drug-drug interactions (DDIs), and adverse drug reactions (ADRs) [9, 10].

In Pakistan, literature reveals a notable incidence of DRPs among CKD patients. One study reported that 86.1% of patients experienced DRPs, with excessive dosing (53.5%) and ADRs (50.5%) being the most common. Similarly, other studies have shown a high prevalence of potential DDIs (84.7%) and inappropriately adjusted antimicrobial doses (71.1%) in CKD patients [11–13]. These concerns not only contribute significantly to morbidity and mortality but also impose a substantial financial burden on the healthcare system [14]. Given the complexity of CKD management and the high risks of medication-related complications, effective care requires a multidisciplinary team of healthcare providers (HCPs), including nephrologists, pharmacists, and nurses [15].

The role of pharmacists is evolving globally from traditional medication dispensing to broader public health and clinical service provision [16–18]. Pharmacists now play an increasingly critical role in patient education, particularly in the management of chronic diseases, where they contribute to counseling, disease prevention, and the coordination of care services [19, 20]. Evidence shows that pharmacist-led interventions in CKD management can yield favorable clinical, humanistic, and economic outcomes. These interventions have been associated with improved treatment adherence, enhanced patient knowledge, better quality of life (QoL), and reduced hospital admissions, DRPs, and emergency department visits [21–24].

This study is centered on the knowledge, attitude, practice, outcome (KAP-O) framework established by Rav-Marathe, Wan, and Marsthe (2016) [25]. The KAP-O model extends traditional KAP theory by outlining a causal pathway in which increased knowledge leads to more favorable attitudes, which, in turn, affect behavioral practices and eventually influence health outcomes. The framework indicates that pharmacists with higher CKD-related knowledge are more inclined to cultivate favorable attitudes towards CKD management, as evidenced in their clinical practices. This study assumes that pharmacists possessing greater knowledge of CKD would exhibit more favorable attitudes, which will correlate with improved practice behaviors, and that these attitudes may mediate the relationship between knowledge and practice.

However, no epidemiological research from Pakistan has yet evaluated pharmacists’ KAP regarding CKD. Addressing this gap, the present study aims to assess pharmacists’ KAP and perceived barriers to CKD-related care in Rawalpindi and Islamabad, Pakistan, and to suggest targeted interventions to improve the overall quality of patient care. The findings from this study will guide the development of specialized educational programs and pharmacist-led interventions. Furthermore, this research seeks to advance patient-centered CKD care by highlighting critical deficiencies, informing future strategies, and identifying specific training needs to strengthen nephrology care in Pakistan.

## Methods

The present study adhered to the STROBE (Strengthening the Reporting of Observational Studies in Epidemiology) checklist [26]. The completed STROBE checklist is available as Additional file 1.

### Study design and setting

A descriptive cross-sectional study was conducted between September 2023 and January 2024, among pharmacists working in Islamabad and Rawalpindi, Pakistan, across public and private healthcare settings.

### Study population

The study population comprised licensed pharmacists working in hospitals and community pharmacies across the study sites. Eligible participants included pharmacists working in public (government-run) or private hospitals, as well as in community pharmacies, with at least 6 months of professional experience. Exclusion criteria included intern pharmacists and pharmacists with less than six months of professional experience.

### Sample size and sampling techniques

According to the Pharmacy Council of Pakistan, the total number of licensed pharmacists in Pakistan as of 2024 was around 59,586 [27]. The sample size was estimated using the Raosoft sample size online calculator [28]. The analytic parameters were selected based on the desired population size, a 5% margin of error, a 95% confidence level (CI), and a 50% non-response rate. The minimum required sample size was estimated to be 382 participants. A total of 432 licensed pharmacists were approached and invited to participate via convenience and snowball sampling. Pharmacists were recruited through direct visits to hospitals and community pharmacies, as well as through professional networks. Snowball sampling was implemented by asking participating pharmacists to recommend eligible colleagues. After obtaining consent and contact information, the researcher contacted the referrer, scheduled a suitable meeting time, and subsequently visited the referrer’s workplace to administer the questionnaire. The techniques were selected based on practical limitations, such as restricted resources, time constraints, and pharmacists’ accessibility, thereby enabling us to recruit a sufficiently large and diverse sample of both hospital and community pharmacists during the study period.

### Development and validation of an instrument

A study instrument was developed based on the Kidney Disease Outcomes Quality Initiative (KDOQI) or Kidney Disease Improving Global Outcome Guidelines (KDIGO) guidelines, and a review of previously published literature on CKD [29–35]. The questionnaire comprised 45 items organized into five sections: demographics, knowledge, attitudes, practices, and barriers. The complete questionnaire is provided as Additional file 2. Section 1 examined the sociodemographic and professional characteristics of the study participants; Sect. 2 assessed pharmacists’ knowledge of CKD; Sect. 3 evaluated pharmacists’ overall attitudes toward CKD care; Sect. 4 focused on practice related to CKD care; and Sect. 5 measured perceived barriers to CKD care.

Before initiating data collection, a preliminary assessment was conducted to evaluate the instrument’s content validity, face validity, and internal consistency. The committee comprised two pharmacy practice researchers and two nephrologists with extensive academic and research expertise. Initially, the research committee reviewed the instrument to ensure the clarity and comprehensibility of the items. The committee advised expanding the attitudes domain to include intention (e.g., willingness to dedicate time for counseling) and self-efficacy (e.g., confidence in communicating with CKD patients) items, which were subsequently integrated to enhance the comprehensiveness of assessing pharmacists’ perceptions and motivational orientation. Their feedback was incorporated to revise the instrument and align it with the study’s objectives.

To assess face validity, a pilot test was conducted with 27 senior pharmacists, each with at least 5 years of professional experience, who were asked to provide feedback on the instrument items, specifically regarding the wording, clarity, and difficulty of the questions. Minor adjustments to the measuring instrument were made in response to their feedback. This specific sample from the pilot study was excluded from the final analysis. The instrument’s internal consistency was assessed using the Statistical Package for the Social Sciences (SPSS) Version 23. The Kuder Richardson Formula 20 (KR-20) value for the knowledge section was 0.762, while Cronbach’s alpha values for the attitude and practice sections were 0.928 and 0.857, respectively. After thorough evaluation and refinement, the finalized questionnaire was used to collect data from the study participants. The questionnaire was therefore developed and administered as an exploratory context-specific instrument to assess CKD-related KAP. Advanced psychometric validation procedures, including exploratory and confirmatory factor analyses and test-retest reliability, were not conducted at this stage because the study was designed as exploratory rather than as a full-scale psychometric development, and due to practical time constraints and the absence of previously validated CKD-KAP instruments.

### Instrument scoring and interpretation

The knowledge section consisted of 15 items, each with response options: “Yes,” “No,” and “Do not know.” A correct response (Yes) awarded one point, while No and Do not Know responses received zero points. The total possible knowledge score ranged from 0 to 15. To categorize knowledge levels, scores were divided into two groups based on the median. A score of 12 or higher was classified as high knowledge, whereas a score of 11 or below was classified as low knowledge. The attitude section included eight items, each measured on a 5-point Likert scale: 1 = Strongly Disagree, 2 = Disagree, 3 = Neutral, 4 = Agree, and 5 = Strongly Agree. The total possible attitude score ranged from 8 to 40. The median score categorized responses into two groups: positive attitude (scores 34–40) and negative attitude (scores 8–33). The practice section consisted of six items and was assessed using a 5-point Likert scale: 1 = Never, 2 = Rarely, 3 = Sometimes, 4 = Most of the time, and 5 = All the time. The total possible score for the practice was 30. Scores were categorized as good practice (≥ 24) and poor practice (≤ 23) based on the median score. Since no validated external benchmarks for CKD KAP scores are available, a median-split approach was employed. This methodology is widely used in KAP research as an effective means to facilitate equitable group comparisons [36–38].

### Data collection procedure

Prior to data collection, the study objectives were clearly explained to all participants. Participation was entirely voluntary, and all pharmacists were assured of confidentiality, anonymity, and their right to withdraw at any stage without consequence. After obtaining informed written consent, participants were provided with a self-administered questionnaire. Completion of the questionnaire took approximately 10 to 15 min. The researcher was available throughout the process to provide clarification when needed.

### Statistical analysis

The principal investigator thoroughly reviewed all collected data to ensure completeness, accuracy, and consistency. Data were coded, entered, and analyzed using SPSS version 23. Descriptive statistics were used to summarize the participants’ demographic characteristics and responses to the questionnaire items. Categorical variables were presented as frequencies and percentages, while continuous variables were summarized using medians and interquartile ranges (IQRs). The Shapiro-Wilk test was used to assess normality. Since the KAP scores were ordinal and not normally distributed, nonparametric tests were used. For group comparisons, the Mann-Whitney U test was used for two-group comparisons and the Kruskal-Wallis test for three or more groups to assess unadjusted differences. The Spearman’s rank correlation coefficient was used to assess the relationship between KAP scores. To identify independent predictors of good knowledge, positive attitude, and good practice, multivariable binary logistic regression analysis was performed.

All sociodemographic and professional characteristics were treated as categorical predictors and entered simultaneously using the enter method, based on theoretical relevance rather than univariate significance, allowing comprehensive adjustment for potential confounding. Binary logistic regression was used because the KAP outcomes were dichotomized and did not meet the assumptions of linear or ordinal regression models. Median-based dichotomization facilitates interpretability in logistic regression. It may reduce score variability and attenuate associations; thus, regression outcomes should be interpreted as comparisons between higher- and lower-KAP groups rather than as absolute competence thresholds. Multicollinearity was assessed using variance inflation factors, which indicated all values below 3, indicating no multicollinearity issues. Model fit was assessed using the Hosmer-Lemeshow goodness-of-fit test (*p* > 0.05), while the Nagelkerke *R*^2^ statistic was used to evaluate the model’s explanatory capacity; exact values are reported in the footnote to Table 4. Results were reported as adjusted odds ratios (AORs) with 95% CIs, and *p*-values < 0.05 were considered statistically significant.

## Results

**Fig. 1:**
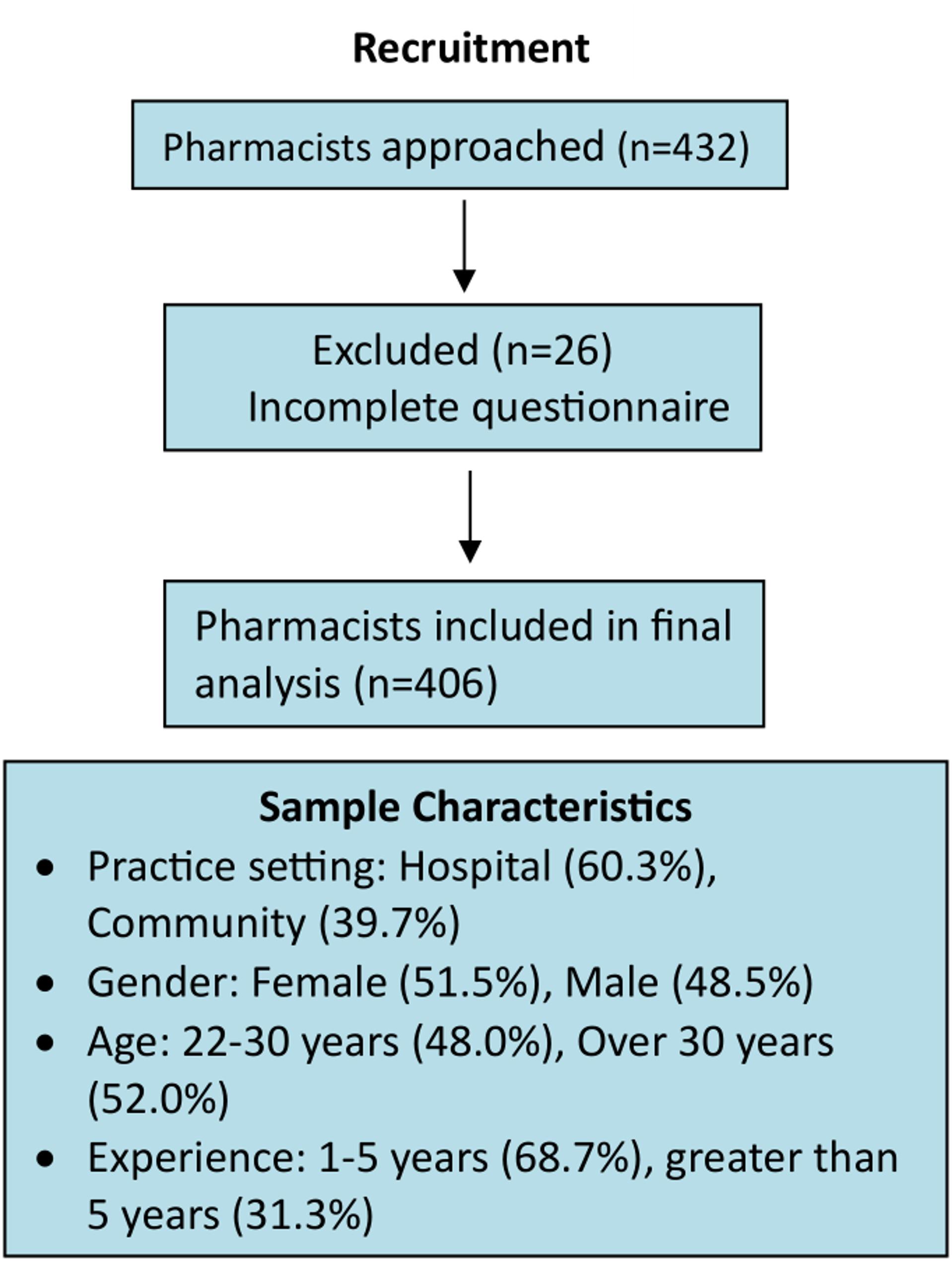
Flowchart of pharmacist recruitment and inclusion in the study

Of the 432 distributed questionnaires, 406 were completed and included in the final analysis, resulting in a response rate of 93.9%. Data from 26 participants were excluded because their questionnaires were incomplete. The participant recruitment and inclusion process is presented in Fig. 1.

### Socio-demographic characteristics of the study participants

The socio-demographic characteristics of the participants are presented in Table 1. More than half were female (*n* = 209, 51.5%), nearly half were aged 22–30 years (*n* = 195, 48.0%), and most held a PharmD degree (*n* = 336, 82.8%). The majority had 1–5 years of experience (*n* = 279, 68.7%), and worked in hospital pharmacies (*n* = 245, 60.3%), primarily in Islamabad (*n* = 264, 65.0%). Nearly half (*n* = 185, 45.6%) reported a monthly income below 50,000 PKR, and over one-third (*n* = 149, 36.7%) reported occasionally encountering CKD patients in their practice.

**Table 1:** Sociodemographic Characteristics of Pharmacists (*N* = 406)

Participants Characteristics	Frequency (%)
Gender	
Female	209 (51.5)
Male	197 (48.5)
Age (Years)	
22–30	195 (48.0)
31–40	166 (40.9)
> 40	45 (11.1)
Qualification	
Pharm-D	336 (82.8)
M.Phil. and PhD	70 (17.2)
Marital status	
Single	281 (69.2)
Married	125 (30.8)
Experience	
1–5	279 (68.7)
6–10	127 (31.3)
Work setting	
Independent community pharmacy	115 (28.3)
Hospital Pharmacy	245 (60.3)
Hospital-affiliated pharmacy	46 (11.4)
Location of Pharmacy	
Islamabad	264 (65.0)
Rawalpindi	142 (35.0)
Monthly Income	
< 50 K	185(45.6)
50–150 K	154(37.9)
> 150 K	67 (16.5)
Weekly Working Hours	
40 h	155(38.2)
48 h	159(39.2)
> 48 h	92(22.7)
How frequently do you encounter patients with chronic kidney disease in your practice?	
Rarely	80 (19.7)
Occasionally	149 (36.7)
Often	131 (32.3)
Very often	46 (11.3)
Have you read the Kidney Disease Improving Global Outcome Guidelines (KDIGO)/Kidney Disease Outcome Quality Initiative (KDOQI) for the management of chronic kidney disease?	
Yes	197 (48.5)
No	209 (51.5)
Have you received any specialized training or education related to chronic kidney disease?	
Yes	106 (26.1)
No	300 (73.9)
Do you have any personal or family experience with chronic kidney disease?	
Yes	114 (28.1)
No	292 (71.9)
Have you ever participated in any chronic kidney disease awareness campaigns or seminars/Conferences?	
Yes	114 (28.1)
No	292 (71.9)
Information Sources?	
Conference & workshops	38 (9.4)
Online medical portal	86 (21.2)
News	36 (8.9)
Online Google	136 (33.5)
Professional journals	50 (12.3)
YouTube	60 (14.8)

Data are presented as frequency (%) unless otherwise specified. Monthly income values are reported in Pakistani Rupees (PKR)

### Pharmacists’ knowledge of chronic kidney disease

Pharmacists’ knowledge of CKD is presented in Supplementary Table 1 (see Additional file 3). The median (IQR) knowledge score was 12 (10–14), with 57.4% of participants demonstrating good knowledge. Most pharmacists correctly identified CKD as a long-term condition (95.6%) and recognized hypertension and diabetes as major risk factors (88.9%). Familiarity with CKD staging criteria was also commonly reported (79.6%).

### Pharmacists’ attitudes toward chronic kidney disease care

Pharmacists’ attitudes towards CKD care are summarized in Supplementary Table 2 (see Additional file 3). The median (IQR) attitude score was 34 (31–37), with 53.0% participants demonstrating a positive attitude. A large majority agreed that community awareness initiatives can improve CKD prevention and management (87.0%), and most acknowledged their professional role in supporting patients with comorbid conditions (84.2%).

### Pharmacists’ practices related to chronic kidney disease care

Pharmacists’ practices related to CKD care are summarized in Supplementary Table 3 (see Additional file 3). The median (IQR) practice score was 24 (21–26), with 50.2% of participants demonstrating good practice. Most pharmacists reported providing ongoing follow-up support to CKD patients (77.6%) and routinely recommending kidney function testing for individuals at high risk of CKD (72.2%).

### Perceived barriers to chronic kidney disease patients’ care

**Fig. 2:**
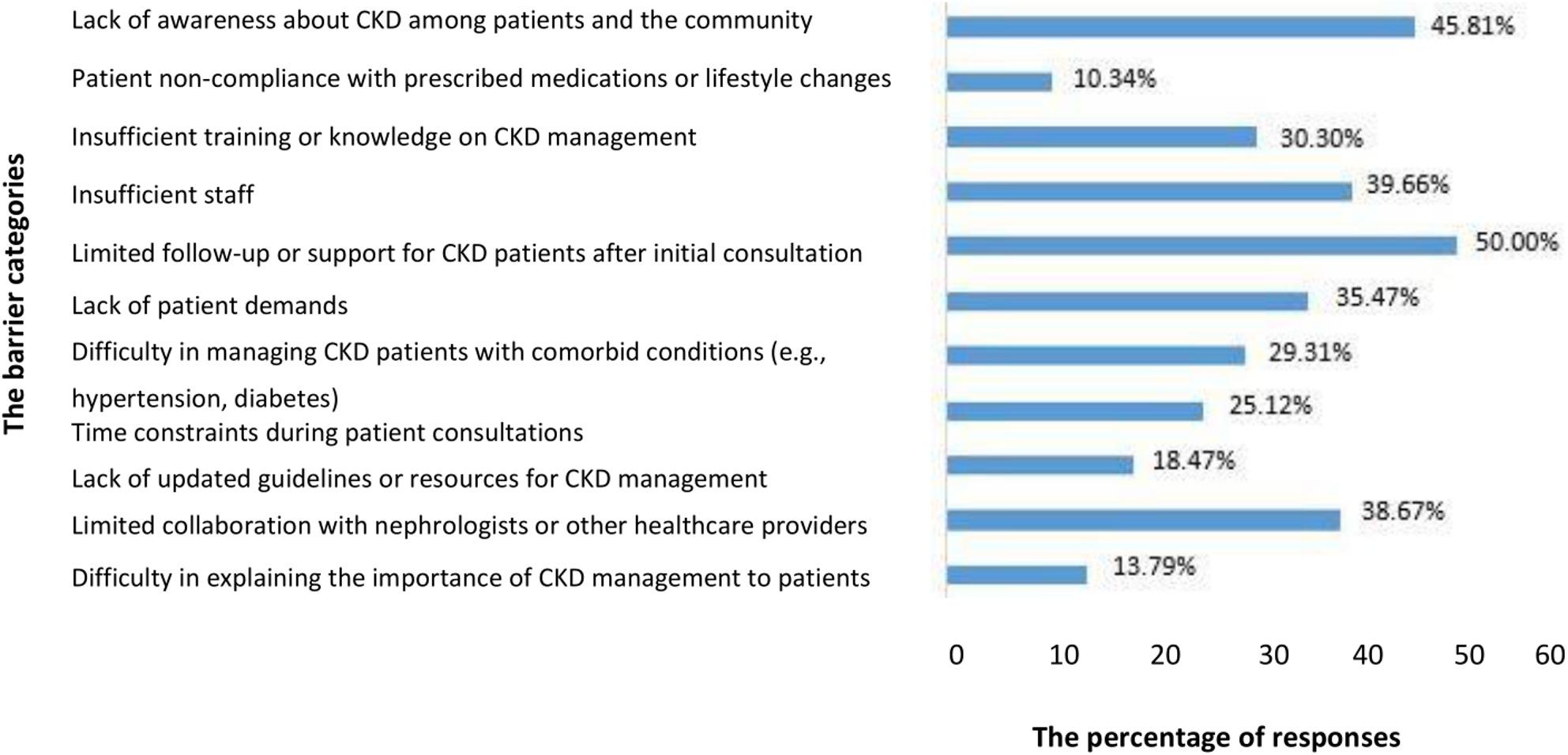
Pharmacist's perceived barriers in chronic kidney disease care (n=406). Note: Bars represent the percentage (%) of respondents reporting each barrier. The x-axis indicates percentage (%) of responses, and the y-axis lists the major perceived barriers

Figure 2 illustrates the perceived barriers to CKD care reported by pharmacists. The most frequently reported barriers were limited follow-up or support for CKD patients after initial consultation (50.0%), lack of awareness about CKD among patients and the community (45.8%), insufficient staffing (39.6%), limited collaboration with nephrologists or other healthcare providers (38.7%), and low patient demands for CKD-related services (35.5%). Less frequently reported barriers included patients’ nonadherence to medication and lifestyle modifications (10.3%) and difficulties explaining CKD management to patients (13.8%).

### Correlation among knowledge, attitude, and practice scores regarding CKD

Table 2 presents the Spearman correlation coefficients assessing the relationships among KAP scores for CKD. A statistically significant positive correlation was found between knowledge and attitude (*r* = 0.207, *p* < 0.001), indicating that higher levels of knowledge were moderately associated with more positive attitudes. Similarly, knowledge was significantly correlated with practice (*r* = 0.237, *p* < 0.001), suggesting that higher knowledge levels were associated with better practice. Notably, a moderate positive correlation was observed between attitudes and practice (*r* = 0.472, *p* < 0.001), indicating that more positive attitudes were associated with better practice behaviors.

**Table 2:** Spearman Correlation Coefficients Between Knowledge, Attitude, and Practice Scores Regarding Chronic Kidney Disease

Variables	Knowledge	Attitude	Practice
Knowledge	**-**		
Attitude	0.207 **(< 0.001)**	**-**	
Practice	0.237 **(< 0.001)**	0.472 **(< 0.001)**	**-**

Values in parentheses indicate *p*-values. All correlations are Spearman’s rank correlation coefficients (r). Two-tailed significance tests were applied; *p* < 0.05 was considered significant

### Comparison of knowledge, attitude, and practice scores among pharmacists according to demographic and professional characteristics

Table 3 presents subgroup comparisons of KAP scores assessed using the Mann-Whitney U and Kruskal-Wallis tests. Knowledge scores were significantly higher among pharmacists who frequently encountered CKD patients than those who encountered CKD less frequently [median (IQR): 14 (11–15) vs. 11 (9–14); *p* < 0.001]. Similarly, pharmacists who reported reading clinical guidelines demonstrated significantly higher knowledge scores than those who did not [13 (10–15) vs. 11 (9–14); *p* < 0.001]. Knowledge scores were also significantly higher among pharmacists who had received CKD-related training [14 (11–15) vs. 12 (9–14); *p* = 0.001] and those who participated in seminars and conferences [13 (10–15) vs. 12 (9.25-14); *p* = 0.017]. Regarding attitude, pharmacists employed in hospital pharmacy settings had significantly higher attitude scores than their counterparts [34 (32–38) vs. 33 (29–36); *p* = 0.023]. In addition, pharmacists practicing in Islamabad demonstrated significantly higher attitude scores than those practicing in Rawalpindi [34 (32-37.75) vs. 32 (30–37); *p* = 0.009]. With respect to practice, pharmacists practicing in Islamabad had significantly higher practice scores than those in Rawalpindi [24 (21–26) vs. 23 (20–24); *p* = 0.004]. Practice scores were also significantly higher among pharmacists who frequently encountered CKD patients than among those who encountered CKD less frequently [24 (22–26) vs. 22 (18–25); *p* = 0.001].

**Table 3:** Comparison of Knowledge, Attitude, and Practice Scores among Pharmacists According to Demographic and Professional Characteristics

Participants Characteristics	Knowledge		Attitude		Practice	
	Median (IQR)	*p*-value	Median (IQR)	*p*-value	Median (IQR)	*p*-value
Gender		0.433		0.310		0.387
Female	12 (10–14)		34 (32–37)		24 (21–26)	
Male	12 (9.50–14)		34 (30–37)		23 (20–26)	
Age (Years)		0.869		0.384		0.263
22–30	12 (10–14)		34 (31–37)		23 (20–26)	
31–40	13 (10-14.25)		34 (31–38)		24 (21–26)	
> 40	12 (8.50–15)		33 (28–37)		24 (20–25)	
Qualification		0.765		0.549		0.599
Pharm-D	12 (10–14)		34 (31–37)		23 (21–26)	
M.Phil. and PhD	12 (8.75-15)		34 (29-37.25)		24 (20.75-26)	
Marital status		0.598		0.447		0.685
Single	12 (10–14)		34 (31–37)		24 (21–26)	
Married	12 (9.50–15)		33 (31-37.50)		24 (21–26)	
Experience		0.951		0.684		0.159
1–5	12 (10–14)		34 (31–37)		23 (21–26)	
6–10	12 (9–15)		34 (30–38)		24 (21–26)	
Work setting		0.466		**0.023**		0.054
Independent community pharmacy	12 (9–15)		33 (29–36)		23 (19–25)	
Hospital Pharmacy	12 (10–14)		34 (32–38)		24 (21-26.50)	
Hospital affiliated pharmacy	11 (9–14)		34 (32–37)		23.50 (20-25.25)	
Location of Pharmacy		0.243		**0.009**		**0.004**
Islamabad	12 (10–14)		34 (32-37.75)		24 (21–26)	
Rawalpindi	12 (9–14)		32 (30–37)		23 (20–24)	
Monthly Income		0.202		0.310		0.690
< 50 K	12 (10–15)		34 (31.50–38)		23 (21–26)	
50–150 K	12 (9–14)		33 (31–37)		24 (21-25.25)	
> 150 K	12 (9–15)		34 (29–37)		24 (20–26)	
Weekly Working Hours		0.453		0.671		0.773
40 h	12 (10–15)		34 (31–37)		23 (20–26)	
48 h	12 (10–14)		34 (31–37)		24 (21–26)	
> 48 h	12 (10–14)		34 (30.25-37)		24 (20.25-25)	
How frequently do you encounter patients with chronic kidney disease in your practice?		**< 0.001**		0.206		**0.012**
Rarely	11 (9–14)		34 (30–37)		22 (18–25)	
Occasionally	12 (9–14)		34 (31–37)		24 (21–26)	
Often	14 (11–15)		35 (31–38)		24 (22–26)	
Very often	12 (9.75–14.25)		34 (30.75-39)		23.5 (19.75–27.25)	
Have you read the Kidney Disease Improving Global Outcome Guidelines (KDIGO)/Kidney Disease Outcome Quality Initiative (KDOQI) for the management of chronic kidney disease?		**< 0.001**		0.249		0.664
Yes	13 (10–15)		33 (30.50–38)		23 (20–26)	
No	11 (9–14)		34 (31.50–37)		24 (21–26)	
Have you received any specialized training or education related to chronic kidney disease?		**0.001**		0.063		0.706
Yes	14 (11–15)		33 (28.75-38)		24 (21–26)	
No	12 (9–14)		34 (31.25-37)		23 (20–26)	
Do you have any personal or family experience with chronic kidney disease?		0.173		0.570		0.421
Yes	13 (10–15)		33 (30–38)		23 (20-25.25)	
No	12 (9.25-14)		34 (31.25-37)		24 (21–26)	
Have you ever participated in any chronic kidney disease awareness campaigns or seminars/Conferences?		**0.017**		**0.005**		0.066
Yes	13 (10–15)		33 (30–38)		23 (20-25.25)	
No	12 (9.25-14)		34 (31.25-37)		24 (21–26)	
Information Sources?		**0.002**		0.443		**< 0.001**
Conference & workshops	13 (11.75-15)		35.5 (31.25-38)		23.5 (20.75-25)	
Online medical portal	13 (10–15)		34 (30–38)		24 (21–27)	
News	10.5 (9–14)		34 (29.25–37.75)		22 (20–24)	
Online Google	11 (9–14)		33 (31–36)		23 (20–25)	
Professional journals	14 (11.75-15)		33 (30.5–38)		25 (23–28)	
YouTube	12 (10–15)		35 (32–37)		23 (20.25–25.75)	

Values are presented as median (interquartile range, IQR). *P*-values were calculated using the Mann–Whitney U test (for two-group comparisons) and the Kruskal–Wallis test (for three or more groups). Statistically significant results (*p* < 0.05) are shown in bold

### Predictors of good knowledge, positive attitude, and good practice among pharmacists

Multivariable binary logistic regression was performed to estimate AORs for predictors of good knowledge, positive attitudes, and good practices, as summarized in Table 4. The findings indicated that participants who received training regarding CKD had significantly higher odds of having good knowledge than those who did not (AOR: 1.934, 95% CI: 1.056–3.543, *p* = 0.033). Regarding attitudes, the results revealed that participants whose pharmacies were located in Islamabad (AOR: 2.096, 95% CI: 1.333–3.297, *p* = 0.001) had significantly higher odds of a positive attitude than those whose pharmacies were located in Rawalpindi. Participants who attended seminars or conferences on CKD had significantly lower odds of reporting a positive attitude than those who did not (AOR: 0.424, 95% CI: 0.243–0.739, *p* = 0.002). In term of practice, pharmacists practicing in Islamabad (AOR: 1.666, 95% CI: 1.054–2.634, *p* = 0.029) and those relying on professional journals as an information source (AOR: 3.510, 95% CI: 1.490–8.268, *p* = 0.004) had significantly higher odds of having a good practice compared to their counterparts in Rawalpindi and those using other information sources.

**Table 4:** Multivariate Binary Logistic Regression Analysis of Predictors of Good Knowledge, Positive Attitude, and Good Practice among Pharmacists in Chronic Kidney Disease Care

Participants Characteristics	Knowledge		Attitude		Practice	
	AOR (95% CI)	*p*-value	AOR (95% CI)	*p*-value	AOR (95% CI)	*p*-value
Gender						
Female	1.274 (0.800–2.030)	0.308	1.062 (0.676–1.668)	0.795	1.235 (0.785–1.942)	0.362
Male	Reference	**-**	Reference	**-**	Reference	**-**
Age (Years)						
22–30	0.666 (0.230–1.932)	0.455	0.855 (0.308–2.378)	0.765	0.688 (0.242–1.950)	0.481
31–40	0.954 (0.381–2.389)	0.919	1.315 (0.545–3.177)	0.542	1.114 (0.452–2.748)	0.814
> 40	Reference	**-**	Reference	**-**	Reference	**-**
Qualification						
Pharm-D	1.424 (0.741–2.737)	0.290	0.802 (0.418–1.536)	0.505	0.855 (0.447–1.636)	0.637
M.Phil. and PhD	Reference	**-**	Reference	**-**	Reference	**-**
Marital status						
Single	1.036 (0.570–1.882)	0.907	1.685 (0.955–2.974)	0.072	1.467 (0.822–2.619)	0.195
Married	Reference	**-**	Reference	**-**	Reference	**-**
Experience						
1–5	1.081 (0.509–2.297)	0.839	0.834 (0.403–1.727)	0.625	0.724 (0.345–1.520)	0.394
6–10	Reference	**-**	Reference	**-**	Reference	**-**
Work setting						
Independent community pharmacy	1.733 (0.805–3.729)	0.160	0.760 (0.358–1.615)	0.476	0.868 (0.408–1.844)	0.712
Hospital Pharmacy	1.449 (0.724–2.898)	0.295	1.072 (0.537–2.142)	0.843	1.390 (0.699–2.765)	0.348
Hospital-affiliated pharmacy	Reference	**-**	Reference	**-**	Reference	**-**
Location of Pharmacy						
Islamabad	1.092 (0.686–1.740)	0.711	2.096 (1.333–3.297)	0.001	1.666 (1.054–2.634)	0.029
Rawalpindi	Reference	**-**	Reference	**-**	Reference	**-**
Monthly Income						
< 50 K	1.803 (0.759–4.284)	0.182	1.067 (0.457–2.487)	0.881	0.699 (0.295–1.675)	0.417
50–150 K	1.236 (0.576–2.653)	0.587	0.779 (0.368–1.651)	0.515	0.784 (0.363–1.692)	0.535
> 150 K	Reference	**-**	Reference	**-**	Reference	**-**
Weekly Working Hours						
40 h	1.510 (0.842–2.709)	0.167	1.164 (0.660–2.051)	0.600	0.796 (0.450–1.409)	0.434
48 h	1.195 (0.672–2.124)	0.545	1.153 (0.660–2.015)	0.616	1.024 (0.584–1.795)	0.934
> 48 h	Reference	**-**	Reference	**-**	Reference	**-**
How frequently do you encounter patients with CKD in your practice?						
Rarely	0.562 (0.239–1.322)	0.187	1.157 (0.497–2.693)	0.735	0.620 (0.268–1.434)	0.264
Occasionally	0.755 (0.349–1.632)	0.474	1.019 (0.480–2.165)	0.960	0.972 (0.461–2.048)	0.940
Often	1.647 (0.755–3.549)	0.210	1.548 (0.729–3.288)	0.255	1.173 (0.559–2.462)	0.672
Very often	Reference	**-**	Reference	**-**	Reference	**-**
Have you read the KDIGO/KDOQI for the management of CKD?						
Yes	1.517 (0.947–2.430)	0.083	0.737 (0.465–1.168)	0.194	0.870 (0.548–1.383)	0.557
No	Reference	**-**	Reference	**-**	Reference	**-**
Have you received any specialized training or education related to CKD?						
Yes	1.934 (1.056–3.543)	0.033	1.011 (0.577–1.771)	0.971	1.606 (0.903–2.855)	0.107
No	Reference	**-**	Reference	**-**	Reference	**-**
Do you have any personal or family experience with CKD?						
Yes	0.919 (0.533–1.582)	0.760	0.904 (0.539–1.518)	0.703	0.715 (0.424–1.205)	0.208
No	Reference	**-**	Reference	**-**	Reference	**-**
Have you ever participated in any CKD awareness campaigns or seminars/Conferences?						
Yes	1.043 (0.585–1.861)	0.886	0.424 (0.243–0.739)	0.002	0.434 (0.246–0.769)	0.004
No	Reference	**-**	Reference	**-**	Reference	**-**
Information Sources?						
Conference & workshops	2.556 (0.955–6.841)	0.062	1.574 (0.630–3.933)	0.331	1.601 (0.661–3.878)	0.297
Online medical portal	1.264 (0.606–2.636)	0.532	0.923 (0.448-1.900)	0.828	1.997 (0.976–4.087)	0.058
News	0.399 (0.156–1.018)	0.055	1.065 (0.423–2.682)	0.894	0.778 (0.307–1.972)	0.597
Online Google	0.659 (0.337–1.289)	0.223	0.550 (0.281–1.075)	0.081	1.004 (0.518–1.946)	0.991
Professional journals	2.097 (0.858–5.125)	0.105	0.478 (0.210–1.092)	0.080	3.510 (1.490–8.268)	0.004
YouTube	Reference	**-**	Reference	**-**	Reference	**-**

Dependent variables were dichotomized using median scores: knowledge (good ≥ 12 vs. poor ≤ 11), attitude (positive ≥ 34 vs. negative ≤ 33), and practice (good ≥ 24 vs. poor ≤ 23). All sociodemographic and professional predictors were included as categorical variables with the last category of each variable was used as the reference group for comparison, as indicated. Multicollinearity was assessed using variance inflation factors (all VIFs < 3). Model fit was evaluated using the Hosmer–Lemeshow goodness-of-fit test (*p* > 0.05). Nagelkerke *R*² values were 0.203 for the knowledge model, 0.142 for the attitude model, and 0.167 for the practice model. Results are presented as adjusted odds ratios (AORs) with 95% confidence intervals (CIs). *p* < 0.05 was considered statistically significant

## Discussion

To our knowledge, this is the first study in Pakistan and among the first globally to comprehensively investigate the KAP of hospital and community pharmacists regarding CKD care. The findings indicate that pharmacists demonstrated moderate KAP regarding CKD care. The observed correlations among KAP domains were statistically significant but modest, suggesting that while knowledge and attitudes are associated with practice, additional contextual factors also play a role. These results are consistent with the global literature among HCPs, including physicians, nurses, and pharmacists, which emphasizes the essential role of HCPs in patient education, counseling, prevention, and disease management [39–43]. Prior international studies also support the effectiveness of pharmacist-led interventions in improving treatment outcomes in CKD [44–46]. However, such causal effects cannot be inferred from the present cross-sectional analysis. Our study provides evidence from a low- and middle-income country setting and suggests potential areas to further strengthen pharmacists’ role in CKD care through targeted, context-specific education and training.

In the knowledge dimension, more than half of the participants (57.4%) had good knowledge. This is comparable to the findings of Sulaiman et al., who reported that 50.9% of pharmacists had adequate knowledge [39]. The considerable proportion with lower knowledge suggests the presence of potential gaps that may warrant further attention. Pharmacists may benefit from access to structured education opportunities and up-to-date, evidence-based resources to ensure they are well equipped to counsel patients with CKD. Participation in formal training opportunities may also support the development of clinical competency [47]. Furthermore, subgroup analysis revealed that pharmacists who frequently encountered patients with CKD exhibited higher knowledge. This finding is consistent with previous research on other chronic diseases, which has shown that regular patient exposure is associated with greater professional awareness and practice [48, 49]. One possible explanation is that frequent contact with patients with CKD provides practical experience that supports familiarity with symptoms and treatment needs; however, this interpretation should be made cautiously, given the cross-sectional design. Pharmacists who attended CKD-related seminars, workshops, and training also showed better knowledge. Similar trends have been reported in other chronic diseases [50, 51]. This association may reflect greater engagement with educational activities, although reverse causality or self-selection cannot be excluded.

In terms of attitudes, just over half of the pharmacists (53.0%) expressed a positive attitude toward CKD care. Subgroup analysis showed that pharmacists working in hospital pharmacies demonstrate a more positive attitude than those employed in community and hospital-affiliated pharmacies. No previous research has directly investigated pharmacists’ attitudes towards CKD, making this a novel contribution. Nonetheless, data from other domains suggest that hospital practice environments may be associated with greater engagement with clinical care. Evidence from different disease areas suggests that hospital pharmacists often have access to structured settings, interdisciplinary collaboration, and clinical resources, which may influence attitudes toward patient care [52]. Community pharmacists, however, face practical barriers, such as limited access to patient records, high dispensing workload, and lack of reimbursement, which may restrict their involvement in expanded clinical roles [53–55]. The findings suggest a potential need for additional support for community pharmacists, particularly through advanced training opportunities within a multidisciplinary care framework and policy initiatives that may help to mitigate identified barriers, thereby fostering favorable attitudes and expanding their roles in CKD management. The inverse association identified between participation in seminars or conferences and a positive attitude warrants careful interpretation. This cross-sectional design does not establish a causal relationship and may be confounded by unmeasured factors or self-selection, whereby pharmacists with less favorable attitudes are more likely to pursue additional educational activities.

For practice, half of the pharmacists (50.0%) showed good performance. Pharmacists with frequent exposure to patients with CKD also reported better practice scores. This association may indicate that repeated patient contact is linked to greater clinical competence and practice behaviour, although causality cannot be established, a pattern also noted by Chiang et al. in asthma care [56]. Furthermore, pharmacists who rely on professional journals also reported better practice, which may reflect greater engagement with evidence-based information. This observation is consistent with previous literature, indicating that engagement with evidence-based resources is associated with improved knowledge and clinical decision-making [57]. In terms of perceived barriers, the most frequently reported challenges to CKD care included limited follow-up after initial consultation (50.0%), lack of awareness among patients and the community (45.8%), insufficient staff (39.6%), limited collaboration with nephrologists or other HCPs (38.7%), and low patient demand for CKD related services (35.5%). Similar barriers have been identified in studies conducted among pharmacists in other regions, including Saudi Arabia [40]. These barriers may reflect perceived systemic and organizational challenges within Pakistan’s healthcare system. Limited follow-up may be related to the absence of structured referral pathways and a tracking mechanism for CKD patients, while inadequate awareness may be influenced by the lack of national CKD screening programs and public health educational initiatives. Staffing shortages and weak collaboration between physicians and pharmacists further highlight potential gaps in healthcare infrastructure and interdisciplinary communication.

Addressing these barriers may require further investigation and consideration by regulatory and policy-level stakeholders, including the establishment of structured follow-up systems, strengthening multidisciplinary care models, and expanding pharmacists’ training in CKD management [58, 59]. These findings must be interpreted within the broader context of Pakistan’s healthcare system. The PharmD curriculum remains largely theoretical, with limited structured clinical clerkship, which may influence graduates’ preparedness for advanced clinical roles. Clinical pharmacy practice continues to evolve, and pharmacists’ involvement in direct patient care remains minimal across both the public and private healthcare sectors [60, 61]. A physician-centric healthcare culture, unclear professional role boundaries, and limited public awareness of pharmacists’ clinical competence may further restrict pharmacists’ participation in patient care. In most settings, pharmacists continue to perform primarily dispensing-related tasks as formal regulatory frameworks that define and support pharmacists’ clinical responsibilities are lacking. Furthermore, collaboration between pharmacists and nephrologists remains limited, as multidisciplinary CKD management models have not yet been fully established in hospitals across Pakistan [62, 63]. Collectively, these contextual factors may help explain the observed finding; however, further longitudinal and interventional research is needed to more clearly assess their impact.

### Strengths and limitations

This study has several strengths, including a rigorous methodology, administration of a standardized and validated questionnaire, and robust statistical analysis, all of which enhance the credibility and validity of the findings. Nevertheless, certain limitations should be acknowledged. First, the cross-sectional design precludes causal inferences, and observed associations between predictors and KAP outcomes should not be interpreted accordingly. Second, the use of convenience and snowball sampling may have introduced selection bias, potentially resulting in an overrepresentation of hospital-based and early-career pharmacists in metropolitan settings and limiting the generalizability of findings beyond Islamabad and Rawalpindi. Third, reliance on self-reported data introduces the possibility of recall and social desirability biases, and residual confounding cannot be entirely excluded. Fourth, the questionnaire was administered as an exploratory, context-specific instrument; although it demonstrated acceptable internal consistency, advanced psychometric validation was not conducted, thereby limiting the ability to confirm construct validity and dimensional structure.

Finally, median-based categorization of KAP scores and the use of yes/no knowledge items may have reduced measurement sensitivity and obscured within-domain variability. Accordingly, the findings should be interpreted as exploratory and hypothesis-generating.

### Practice implications and future studies

The findings of this study highlight the need for structured, ongoing educational programs focused on treatment, diets, and lifestyle changes, as well as training in patient-centered care and communication skills, to enhance pharmacists’ knowledge levels and clinical competencies in CKD care. It can contribute to the early identification of CKD, facilitate better patient counseling, reduce DRPs, and improve patient adherence and QOL. This is supported by several studies that have shown pharmacists who participated in learning activities and training used the information more extensively and were more involved in-patient care for those with CKD, positively impacting the patients [64–67]. Additionally, encouraging teamwork among HCPs is essential in developing a shared approach to managing CKD-related challenges. Interdisciplinary training and collaboration can enhance attitudes by fostering a collective comprehension of the difficulties involved.

Further studies should employ probability sampling techniques and longitudinal designs to enhance generalizability and facilitate causal inference. In addition, large-scale psychometric validation, including CVI/CVR calculation, factor analysis, and test-retest reliability, is recommended to enhance the tool’s reliability and validity. Qualitative studies should be conducted from both pharmacists’ and patients’ perspectives to enhance representativeness and depth, thereby informing the development of the best pharmacy education program and addressing its needs. Moreover, future research should investigate the long-term effects of educational interventions on pharmacists and the impact of pharmacist-led interventions on patient outcomes. Such evidence would ultimately contribute to the development of more effective self-management strategies for patients with CKD.

## Conclusions

Gaps exist in pharmacists’ CKD-related KAP, highlighting the need for targeted education and training programs. Strengthening pharmacy curricula and providing ongoing professional development are crucial to enhancing pharmacists’ clinical competencies and improving CKD care. To address these challenges, it is essential to implement comprehensive training programs, encourage ongoing professional development, and promote collaboration between pharmacists and HCPs. Additionally, policymakers should support continuing education programs that equip pharmacists with up-to-date guidelines for CKD management, treatment strategies, and patient counseling techniques.

## Supplementary Information

Supplementary Material 1.

Supplementary Material 2.

Supplementary Material 3.

## Data Availability

The datasets generated and/or analyzed during the current study are available from the corresponding author upon request.

## Abbreviations

ADR: Adverse Drug Reaction
AOR: Adjusted Odds Ratio
CI: Confidence Interval
CKD: Chronic Kidney Disease
CVI: Content Validity Index
CVR: Content Validity Ratio
DDIs: Drug-Drug Interactions
DRP: Drug-Related Problem
HCPs: Healthcare providers
IQR: Interquartile Range
KAP: Knowledge, Attitude, and Practice
KDIGO: Kidney Disease: Improving Global Outcomes
KDOQI: Kidney Disease Outcomes Quality Initiative
QoL: Quality of Life
SD: Standard Deviation
SPSS: Statistical Package for the Social Sciences
STROBE: Strengthening the Reporting of Observational Studies in Epidemiology
