# Supplementary material for: Knowledge, attitudes, and practices of pharmacists towards chronic kidney disease in Rawalpindi and Islamabad, Pakistan: a cross-sectional study
Source: BMC Med Educ. 2026 Mar 24;26:711. doi: 10.1186/s12909-026-09070-5 (PMC13137725; doi:10.1186/s12909-026-09070-5)
Supplement: Supplementary file 2 — Supplementary Material 2. [file 12909_2026_9070_MOESM2_ESM.docx]

Knowledge, Attitudes, and Practices of Pharmacists Toward Chronic Kidney Disease in Pakistan: A Cross-Sectional Study

INFORMED CONSENT FORM:

Assalamu alaikum, you are kindly invited to take part in a study focused on the "Knowledge, Attitudes, and Practices of Pharmacists Towards Chronic Kidney Disease. The objective of this study is to gain a deeper understanding of pharmacists' perspectives and their engagement with chronic kidney disease. Your participation is vital and entirely voluntary. Your responses will be treated confidentially and used solely for research purposes. Your contribution to this study is highly appreciated.

Participant's Signature: Date:

TOOL FOR DATA COLLECTION Section 1: Demographic Information

1. **Name (Optional):**
2. **Gender:** ☐ F ☐ M
3. **Age (Years):** ☐ 22-30 ☐ 31-40 ☐ >41
4. **Educational qualification:** ☐ PharmD ☐ MPhil ☐ PhD
5. **Marital status:** ☐ Single ☐ Married
6. **Experience (Years):** ☐ 1-5 ☐ 6–10 ☐ >10
7. **Work Setting:** ☐ Independent Community Pharmacy ☐ Hospital Pharmacy
   - Hospital-affiliated Pharmacy
8. **Location of pharmacy:** ☐ Islamabad ☐ Rawalpindi
9. **Monthly income:** ☐ <50K ☐ 50K-150K ☐ >150K
10. **Your average working hours per week:** ☐ 40 hours ☐ 48 hours ☐ > 48 hours
11. **How frequently do you encounter patients with chronic kidney disease in your practice?**
    - Rarely ☐ Occasionally ☐ Often ☐ Very often
12. **Have you read the Kidney Disease Improving Global Outcome Guidelines (KDIGO)/Kidney Disease Outcome Quality Initiative (KDOQI) for the management of chronic kidney disease?**
    - Yes ☐ No
13. **Have you received any specialized training or education related to chronic kidney disease?**
    - Yes ☐ No
14. **Do you have any personal or family experience with chronic kidney disease?**
    - Yes ☐ No
15. **Have you ever participated in any chronic kidney disease awareness campaigns or seminars/Conferences?**
    - Yes ☐ No
16. **What are your information sources?**
    - Conferences & Workshops ☐ Online Medical Portal ☐ News
    - Professional Journals ☐ YouTube ☐ Online Resources (e.g., Google)

Section 2: Knowledge of Chronic Kidney Disease Among Pharmacists (Yes, No, Don't Know)

1. **Chronic kidney disease is a long-term condition that affects the normal functioning of the kidneys.**
   - Yes ☐ No ☐ Don't Know
2. **Are you familiar with the five stages of chronic kidney disease, and each stage has its management plan to help reduce complications and slow down CKD progression?**
   - Yes ☐ No ☐ Don't Know
3. **Do you know the recommended blood pressure targets for patients with chronic kidney disease?**
   - Yes ☐ No ☐ Don't Know
4. **Hypertension and diabetes are common risk factors for chronic kidney disease.**
   - Yes ☐ No ☐ Don't Know
5. **Are you aware of the common medications that can potentially worsen kidney function?**
   - Yes ☐ No ☐ Don't Know
6. **Chronic kidney disease can be diagnosed through blood and urine tests.**
   - Yes ☐ No ☐ Don't Know
7. **Are you knowledgeable about the potential drug dosing adjustments needed for patients with reduced kidney function?**
   - Yes ☐ No ☐ Don't Know
8. **Are you familiar with the key differences between acute kidney injury and chronic kidney disease?**
   - Yes ☐ No ☐ Don't Know
9. **Late referral to a nephrologist causes high morbidity, mortality and rate of hospitalization in CKD patients.**
   - Yes ☐ No ☐ Don't Know
10. **Are you aware of the potential interactions between common over-the-counter medications and chronic kidney disease medications?**
    - Yes ☐ No ☐ Don't Know
11. **Are you knowledgeable about the impact of chronic kidney disease on drug metabolism and clearance?**
    - Yes ☐ No ☐ Don't Know
12. **Do you know any standard treatment guidelines for CKD management?**
    - Yes ☐ No ☐ Don't Know
13. **Do you understand the concept of glomerular filtration rate (GFR) and its significance in chronic kidney disease management?**
    - Yes ☐ No ☐ Don't Know
14. **Do you understand the role of angiotensin-converting enzyme inhibitors (ACE inhibitors) and angiotensin II receptor blockers (ARBs) in chronic kidney disease management?**
    - Yes ☐ No ☐ Don't Know
15. **Is eGFR (estimated Glomerular Filtration Rate) a better indicator of chronic kidney disease severity than serum creatinine alone?**
    - Yes ☐ No ☐ Don't Know

Section 3: Pharmacists' Attitudes Toward Chronic Kidney Disease Care (Strongly Disagree, Disagree, Neutral, Agree, Strongly Agree)

1. **I believe that CKD education and awareness are important aspects of my role as a pharmacist.**
   - Strongly Disagree ☐ Disagree ☐ Neutral ☐ Agree ☐ Strongly Agree
2. **I feel confident in my ability to effectively communicate with chronic kidney disease patients about their medications and treatment plans.**
   - Strongly Disagree ☐ Disagree ☐ Neutral ☐ Agree ☐ Strongly Agree
3. **I believe that early detection and intervention for chronic kidney disease can significantly improve patients' quality of life.**
   - Strongly Disagree ☐ Disagree ☐ Neutral ☐ Agree ☐ Strongly Agree
4. **I am willing to dedicate extra time to counsel chronic kidney disease patients on lifestyle modifications and medication adherence.**
   - Strongly Disagree ☐ Disagree ☐ Neutral ☐ Agree ☐ Strongly Agree
5. **I believe that raising awareness about chronic kidney disease in the community can lead to better prevention and management of the disease.**
   - Strongly Disagree ☐ Disagree ☐ Neutral ☐ Agree ☐ Strongly Agree
6. **I am concerned about the financial burden that CKD treatment places on patients.**
   - Strongly Disagree ☐ Disagree ☐ Neutral ☐ Agree ☐ Strongly Agree
7. **I believe that pharmacists play a significant role in helping chronic kidney disease patients understand and manage their comorbid conditions.**
   - Strongly Disagree ☐ Disagree ☐ Neutral ☐ Agree ☐ Strongly Agree
8. **I am committed to staying up-to-date with the latest advancements and guidelines in chronic kidney disease management to provide the best care.**
   - Strongly Disagree ☐ Disagree ☐ Neutral ☐ Agree ☐ Strongly Agree

Section 4: Pharmacists' Practices in Chronic Kidney Disease Care (Very-Unlikely, Unlikely, Neutral, Likely, Very-Likely)

1. **How likely are you to include chronic kidney disease-specific medication counselling in your patient interactions?**
   - Very Unlikely ☐ Unlikely ☐ Neutral ☐ Likely ☐ Very Likely
2. **How likely are you to collaborate with nephrologists for medication adjustments in chronic kidney disease patients?**
   - Very Unlikely ☐ Unlikely ☐ Neutral ☐ Likely ☐ Very Likely
3. **How likely are you to recommend OTC medications that are safe for chronic kidney disease patients?**
   - Very Unlikely ☐ Unlikely ☐ Neutral ☐ Likely ☐ Very Likely
4. **How likely are you to advocate for routine kidney function testing in high-risk patient populations?**
   - Very Unlikely ☐ Unlikely ☐ Neutral ☐ Likely ☐ Very Likely
5. **How likely are you to provide ongoing support and follow-up to CKD patients for their medication and health needs?**
   - Very Unlikely ☐ Unlikely ☐ Neutral ☐ Likely ☐ Very Likely
6. **How likely are you to refer patients with CKD symptoms to a nephrologist?**
   - Very Unlikely ☐ Unlikely ☐ Neutral ☐ Likely ☐ Very Likely

Section 5: Barriers in Chronic Kidney Disease (CKD) Care

What are the main barriers you face in chronic kidney disease (CKD) patients' care? (Please select all that apply.)

☐ Lack of updated guidelines or resources for CKD management

☐ Time constraints during patient consultations

☐ Insufficient training or knowledge on CKD management

☐ Limited collaboration with nephrologists or other healthcare providers

☐ Difficulty in explaining the importance of CKD management to patients

☐ Patient non-compliance with prescribed medications or lifestyle changes

☐ Limited follow-up or support for CKD patients after initial consultation

☐ Difficulty in managing CKD patients with comorbid conditions (e.g., hypertension, diabetes)

☐ Lack of awareness about CKD among patients and the community

☐ Lack of patient demands

☐ Insufficient staff

THE END

Any Feedback/Suggestions:

____________________________________________________________________________________________________________

____________________________________________________________________________________________________________

____________________________________________________________________________________________________________

__________________________________________________________________________________________

__________________________________________________________________________________________

"Your willingness to contribute to my study is deeply appreciated. Thank you!"
