# Supplementary material for: Knowledge, attitudes, and practices of pharmacists towards chronic kidney disease in Rawalpindi and Islamabad, Pakistan: a cross-sectional study
Source: BMC Med Educ. 2026 Mar 24;26:711. doi: 10.1186/s12909-026-09070-5 (PMC13137725; doi:10.1186/s12909-026-09070-5)
Supplement: Supplementary file 3 — Supplementary Material 3. [file 12909_2026_9070_MOESM3_ESM.docx]

**Supplementary Table 1. Item-wise Responses to Knowledge Questions on Chronic Kidney Disease Among Pharmacists (N = 406)**

| **Knowledge Questions** | | **Yes**  **n (%)** | **No**  **n (%)** | **Don't know**  **n (%)** |
| --- | --- | --- | --- | --- |
| **Chronic kidney disease is a long-term condition that affects the normal functioning of the kidneys.** | | 388 (95.6) | 7 (1.7) | 11 (2.7) |
| **Are you familiar with the** **five stages of chronic kidney disease, and each stage has its own management plan to help reduce complications and slow down the progression of chronic kidney disease?** | | 323 (79.6) | 56 (13.8) | 27 (6.7) |
| **Do you know the recommended blood pressure targets for patients with chronic kidney disease?** | | 296 (72.9) | 66 (16.3) | 44 (10.8) |
| **Hypertension and diabetes are common risk factors for chronic kidney disease.** | | 361 (88.9) | 19 (4.7) | 26 (6.4) |
| **Are you aware of the common medications that can potentially worsen kidney function?** | | 327 (80.5) | 41 (10.1) | 38 (9.4) |
| **Chronic kidney disease can be diagnosed through blood and urine tests.** | | 351 (86.5) | 18 (4.4) | 37 (9.1) |
| **Are you knowledgeable about the potential drug dosing adjustments needed for patients with reduced kidney function?** | | 280 (69.0) | 75 (18.5) | 51 (12.6) |
| **Are you familiar with the key differences between acute kidney injury and chronic kidney disease?** | | 336 (82.8) | 40 (9.9) | 30 (7.4) |
| **Late referral to a nephrologist causes high morbidity, mortality, and rate of hospitalization in CKD patients.** | | 332 (81.8) | 39 (9.6) | 35 (8.6) |
| **Are you aware of the potential interactions between common over-the-counter medications and chronic kidney disease medications?** | | 294 (72.4) | 55 (13.5) | 57 (14.0) |
| **Are you knowledgeable about the impact of chronic kidney disease on drug metabolism and clearance?** | | 233 (57.4) | 109 (26.8) | 64 (15.8) |
| **Do you know any standard treatment guidelines for CKD management?** | | 322 (79.3) | 44 (10.8) | 40 (9.9) |
| **Do you understand the concept of glomerular filtration rate (GFR) and its significance in chronic kidney disease management?** | | 352 (86.7) | 26 (6.4) | 28 (6.9) |
| **Do you understand the role of angiotensin-converting enzyme inhibitors (ACE inhibitors) and angiotensin II receptor blockers (ARBs) in chronic kidney disease management?** | | 312 (76.8) | 55 (13.5) | 39 (9.6) |
| **Is eGFR (estimated Glomerular Filtration Rate) a better indicator of chronic kidney disease severity than serum creatinine alone?** | | 263 (64.8) | 83 (20.4) | 60 (14.8) |
| **Overall, Knowledge** | **Mean (SD)**  **Median (IQR)** | 11.7 (2.93)  12 (10-14) | | |
| **Knowledge Classification** | **Low Knowledge (0-11)**  **High Knowledge (12-15)** | 173 (42.6)  233 (57.4) | | |

**Note:** Knowledge responses were coded as “Yes” = 1 point and “No/Don’t know” = 0 points. Data are presented as frequency (%).

**Supplementary Table 2. Item-wise Responses to Attitude Statements on Chronic Kidney Disease Care Among Pharmacists (N = 406)**

| **Attitude questions** | | **Strongly Disagree**  **n (%)** | **Disagree**  **n (%)** | **Neutral**  **n (%)** | **Agree**  **n (%)** | **Strongly Agree**  **n (%)** |
| --- | --- | --- | --- | --- | --- | --- |
| **I believe that CKD education and awareness are important aspects of my role as a pharmacist.** | | 37 (9.1) | 5 (1.2) | 33 (8.1) | 141 (34.7) | 190 (46.8) |
| **I feel confident in my ability to effectively communicate with chronic kidney disease patients about their medications and treatment plans.** | | 23 (5.7) | 11 (2.7) | 81 (20.0) | 186 (45.8) | 105 (25.9) |
| **I believe that early detection and intervention for chronic kidney disease can significantly improve a patient's quality of life.** | | 28 (6.9) | 5 (1.2) | 38 (9.4) | 152 (37.4) | 183 (45.1) |
| **I am willing to dedicate extra time to counsel chronic kidney disease patients on lifestyle modifications and medication adherence.** | | 15 (3.7) | 3 (0.7) | 53 (13.1) | 192 (47.3) | 143 (35.2) |
| **I believe that raising awareness about chronic kidney disease in the community can lead to better prevention and management of the disease.** | | 20 (4.9) | 7 (1.7) | 26 (6.4) | 166 (40.9) | 187 (46.1) |
| **I am concerned about the financial burden that CKD treatment places on patients.** | | 15 (3.7) | 11 (2.7) | 55 (13.5) | 183 (45.1) | 142 (35.0) |
| **I believe that pharmacists play a significant role in helping chronic kidney disease patients understand and manage their comorbid conditions.** | | 22 (5.4) | 5 (1.2) | 37 (9.1) | 160 (39.4) | 182 (44.8) |
| **I am committed to staying up-to-date with the latest advancements and guidelines in chronic kidney disease management to provide the best care.** | | 15 (3.7) | 4 (1.0) | 44 (10.8) | 186 (45.8) | 157 (38.7) |
| **Overall Attitude** | **Mean (SD)**  **Median (IQR)** | 32.7 (6.57)  34 (31-37) | | | | |
| **Attitude Classification** | **Negative Attitude (1-33)**  **Positive Attitude (34-40)** | 191 (47.0)  215 (53.0) | | | | |

**Note:** Attitude items were rated on a 5-point Likert scale (1 = Strongly Disagree to 5 = Strongly Agree). Data are presented as frequency (%).

**Supplementary Table 3. Item-wise Responses to Practice Behaviors Related to Chronic Kidney Disease Care Among Pharmacists (N = 406)**

| **Practice questions** | | **Very Unlikely**  **n (%)** | **Unlikely**  **n (%)** | **Neutral**  **n (%)** | **Likely**  **n (%)** | **Very Likely**  **n (%)** |
| --- | --- | --- | --- | --- | --- | --- |
| **How likely are you to include chronic kidney disease-specific medication counselling in your patient interactions?** | | 14 (3.4) | 32 (7.9) | 65 (16.0) | 211 (52.0) | 84 (20.7) |
| **How likely are you to collaborate with nephrologists for medication adjustments in chronic kidney disease patients?** | | 17 (4.2) | 54 (13.3) | 68 (16.7) | 166 (40.9) | 101 (24.9) |
| **How likely are you to recommend OTC medications that are safe for chronic kidney disease patients?** | | 12 (3.0) | 48 (11.8) | 74 (18.2) | 177 (43.6) | 95 (23.4) |
| **How likely are you to advocate for routine kidney function testing in high-risk patient populations?** | | 8 (2.0) | 38 (9.4) | 67 (16.5) | 190 (46.8) | 103 (25.4) |
| **How likely are you to provide ongoing support and follow-up to CKD patients for their medication and health needs?** | | 8 (2.0) | 30 (7.4) | 53 (13.1) | 205 (50.5) | 110 (27.1) |
| **How likely are you to refer patients with CKD symptoms to a nephrologist?** | | 9 (2.2) | 39 (9.6) | 63 (15.5) | 181 (44.6) | 114 (28.1) |
| **Overall Practice** | **Mean (SD)**  **Median (IQR)** | 22.8 (4.61)  24 (21-26) | | | | |
| **Practice Classification** | **Poor Practice (1-23)**  **Good Practice (24-30)** | 202 (49.8)  204 (50.2) | | | | |

**Note:** Practice items were rated on a 5-point Likert scale (1 = Very Unlikely to 5 = Very Likely). Data are presented as frequency (%).
